# Genetic and pharmacological evidence that G2019S LRRK2 confers a hyperkinetic phenotype, resistant to motor decline associated with aging

**DOI:** 10.1016/j.nbd.2014.07.013

**Published:** 2014-11

**Authors:** Francesco Longo, Isabella Russo, Derya R. Shimshek, Elisa Greggio, Michele Morari

**Affiliations:** a Department of Medical Sciences, Section of Pharmacology, and National Institute of Neuroscience, University of Ferrara, via Fossato di Mortara 17-19, 44121 Ferrara, Italy; b Department of Biology, University of Padova, Via Ugo Bassi 58/B, 35131 Padova, Italy; c Department of Neuroscience, Novartis Institutes for BioMedical Research, Novartis Pharma AG, 4002 Basel, Switzerland

**Keywords:** BAC, bacterial artificial chromosome, DA, dopamine, KI, knock-in, KD, kinase dead, LRRK2, leucine-rich repeat kinase 2, PD, Parkinson's disease, ROC, Ras Of Complex, WT, wild-type, Aging, D1994S knock-in, G2019S knock-in, H-1152, LRRK2 kinase-dead, LRRK2, LRRK2 kinase inhibitors, Nov-LRRK2-11, Parkinson's disease, Ser935 phosphorylation

## Abstract

The leucine-rich repeat kinase 2 mutation G2019S in the kinase-domain is the most common genetic cause of Parkinson's disease. To investigate the impact of the G2019S mutation on motor activity in vivo, a longitudinal phenotyping approach was developed in knock-in (KI) mice bearing this kinase-enhancing mutation. Two cohorts of G2019S KI mice and wild-type littermates (WT) were subjected to behavioral tests, specific for akinesia, bradykinesia and overall gait ability, at different ages (3, 6, 10, 15 and 19 months). The motor performance of G2019S KI mice remained stable up to the age of 19 months and did not show the typical age-related decline in immobility time and stepping activity of WT. Several lines of evidence suggest that enhanced LRRK2 kinase activity is the main contributor to the observed hyperkinetic phenotype of G2019S KI mice: i) KI mice carrying a LRRK2 kinase-dead mutation (D1994S KD) showed a similar progressive motor decline as WT; ii) two LRRK2 kinase inhibitors, H-1152 and Nov-LRRK2-11, acutely reversed the hyperkinetic phenotype of G2019S KI mice, while being ineffective in WT or D1994S KD animals. LRRK2 target engagement in vivo was further substantiated by reduction of LRRK2 phosphorylation at Ser935 in the striatum and cortex at efficacious doses of Nov-LRRK2-11, and in the striatum at efficacious doses of H-1152. In summary, expression of the G2019S mutation in the mouse *LRRK2* gene confers a hyperkinetic phenotype that is resistant to age-related motor decline, likely via enhancement of LRRK2 kinase activity. This study provides an in vivo model to investigate the effects of LRRK2 inhibitors on motor function.

## Highlights

• The LRRK2 G2019S mutation confers a hyperkinetic phenotype.
• The LRRK2 D1994S kinase-dead mutation does not affect motor phenotype.
• The LRRK2 kinase inhibitors reverse motor phenotype of G2019S mice.
• The LRRK2 kinase inhibitors inhibit LRRK2 phosphorylation at Ser935 ex-vivo.

## Introduction

Mutations in the leucine-rich repeat kinase 2 (*LRRK2*) gene (PARK8, OMIM 609007) are associated to late-onset, autosomal dominant Parkinson's disease (PD), and account for up to 13% of familial and 1–2% of sporadic PD cases (Paisan-Ruiz et al., 2004, Zimprich et al., 2004). LRRK2-associated PD is clinically similar to idiopathic forms, and is characterized by the degeneration of *substantia nigra* dopaminergic neurons usually with α-synuclein and ubiquitin positive Lewy body formation (Healy et al., 2008). Furthermore, variations in LRRK2 have been linked to other diseases, leprosy (Zhang et al., 2009), cancer (Hassin-Baer et al., 2009) and possibly inflammatory bowel disease (Barrett et al., 2008) although the latter is controversial (Kumar et al., 2013). LRRK2 is a large multifunctional protein, essentially consisting of a GTPase/ROC (Ras Of Complex) along with its COR (C-terminal Of ROC) domain, a kinase domain, and a number of protein-protein interaction domains including ankyrin and leucine-rich repeat motifs at the N-terminus, and WD40 repeats at the C-terminus (Cookson, 2010, Marin, 2006). The pathogenic mutations of LRRK2 are clustered among the central tridomain region that forms the catalytic core of the protein (Cookson, 2010, Mata et al., 2006). The substitution of a glutamate with a serine in position 2019 (G2019S) is the most common familial mutation, and has attracted greater interest because it enhances LRRK2 kinase activity in vitro (Greggio et al., 2006, Jaleel et al., 2007, West et al., 2005) and in vivo (Sheng et al., 2012), resulting in neuronal toxicity in vitro (Iaccarino et al., 2007, Smith et al., 2005). Interestingly, non-selective LRRK2 inhibitors were shown to protect against G2019S LRRK2-induced neurodegeneration in vivo (Liu et al., 2011), indicating that inhibition of LRRK2 activity may represent a valuable target in a PD therapeutic perspective. Accordingly, these findings have provided the rationale for developing selective LRRK2 kinase inhibitors (Choi et al., 2012, Estrada et al., 2012, Estrada et al., 2014, Herzig et al., 2011, Nichols et al., 2009, Troxler et al., 2013) for their potential antiparkinsonian activity (Lee et al., 2010, Liu et al., 2011).

Quite disappointingly, however, the attempts to reproduce parkinsonian-like motor deficits in rodents expressing G2019S LRRK2 have led to inconsistent results (for recent reviews see: Yue and Lachenmayer, 2011), and, as a consequence, a reliable rodent model for testing motor effects of LRRK2 inhibitors in vivo is currently unavailable. Indeed, mice overexpressing human or murine G2019S using bacterial artificial chromosome (BAC) transgenesis did not show any impairment of motor performance, and instead were found hyperactive in some tests (Li et al., 2010, Melrose et al., 2010). Consistently, mice overexpressing human G2019S LRRK2 under the Thy1 (Herzig et al., 2012), CaMKII (TetO) (Lin et al., 2009) or CMV/PDGF (Ramonet et al., 2011) artificial promoters showed, if any, improvements in motor activity. Finally, rats temporarily (but not constitutively) overexpressing G2019S, show increased exploratory behavior in the open field at 20 months but not at earlier ages (Zhou et al., 2011). Although it is possible that the degree of G2019S transgene overexpression in midbrain dopamine (DA) neurons, which is promoter-dependent, drives the motor phenotype (Chen et al., 2012), the data so far accumulated in rodents overexpressing G2019S LRRK2 suggest, at most, that low expression levels of G2019S are not detrimental for motor function. Actually, the consistent observations of test-dependent, mild improvements of motor activity across these studies call for a more in-depth analysis of the impact of G2019S LRRK2 on motor function, using a longitudinal phenotyping strategy and behavioral tests more specific for motor function. In fact, most studies are limited to the use of the open field test, where motor performance can be influenced by affective states. In addition, studies in G2019S overexpressing animals may be criticized for artificially enhancing LRRK2 levels in areas where LRRK2 physiological expression is low (e.g. the *substantia nigra pars compacta*), and for overlooking the interference between LRRK2 mutants and native endogenous LRRK2, still expressed.

For these reasons, in the present study we enrolled two cohorts of G2019S knock-in (KI) mice and wild-type littermates (WT), and analyzed their motor activity from the age of 3 to 19 months, using a set of complementary behavioral tests, specific for akinesia, bradykinesia and overall gait ability (the bar, drag and rotarod tests; Marti et al., 2005, Viaro et al., 2008). Our study revealed that G2019S KI mice had enhanced motor activity compared to WT already at 3 months of age, and throughout aging. To confirm that enhanced kinase activity accounts for this phenotype, we performed a parallel longitudinal study in mice carrying a LRRK2 mutation (D1994S) that impairs kinase activity (kinase-dead, D1994S KD), in comparison with their own WT. In addition, we tested the ability of small molecular-weight ATP analogous LRRK2 kinase inhibitors to reverse the hyperkinetic phenotype of G2019S KI mice. In vivo LRRK2 targeting of kinase inhibitors was confirmed by measuring LRRK2 phosphorylation at Ser935 (Dzamko et al., 2010).

## Materials and methods

### Animals

Male homozygous LRRK2 G2019S KI and KD mice backcrossed on a C57Bl/6J background were obtained from Novartis Institutes for BioMedical Research, Novartis Pharma AG (Basel, Switzerland). Male non-transgenic wild-type (WT) mice were littermates obtained from the respective heterozygous breeding. The mice used in our study were generated at Novartis laboratories, and were previously characterized from several biochemical and neuropathological standpoints, although motor analysis was limited to the open field in 5-month old animals (Herzig et al., 2011).

Mice employed in the study were kept under regular lighting conditions (12 h light/dark cycle) and given food and water ad libitum. Experimental procedures involving the use of animals were approved by the Ethical Committee of the University of Ferrara and the Italian Ministry of Health (licenses 171/2010-B and 318/2013-B). Adequate measures were taken to minimize animal pain and discomfort.

### Experimental design

The longitudinal study was conducted on two cohorts of G2019S KI (n = 19) and their WT (n = 12) littermates. Mice were received from Novartis at the age of about 2 months, and accommodated in the vivarium of the University of Ferrara. Mice were subjected to motor tests at 3, 6, 10, 15 and 19 months. D1994S KD and their WT mice (n = 10 each) were tested at 3, 6, 10 and 15 months. Separate, age-matched cohorts of 3, 10, 14 and 18-month-old WT and G2019S KI mice (6–8 mice per group) were enrolled in transversal behavioral studies, to parallel the results of the longitudinal study. Finally, other cohorts of mice (n = 10 each genotype) were used for pharmacological studies with LRRK2 kinase inhibitors at 6, 12 and 15 months (G2019S KI), or 12 months (D1994S KD).

### Behavioral tests

Motor activity was evaluated by means of three behavioral tests specific for different motor abilities, as previously described (Marti et al., 2005, Marti et al., 2007, Viaro et al., 2008): the bar, drag and rotarod test. Animals were trained for 4 days to the specific motor tasks in order to obtain a reproducible motor response, and then tested at the 5th day, both in the phenotyping and pharmacological studies. For pharmacological studies, the test sequence (bar, drag and rotarod) was repeated before (T0) and at different time-points (depending on the experiment) after drug injection. Experimenters were blinded to genotype and treatments.

### Bar test

Originally developed to quantify morphine-induced catalepsy (Kuschinsky and Hornykiewicz, 1972), this test measures the ability of the animal to respond to an externally imposed static posture. Also known as the catalepsy test (for a review see (Sanberg et al., 1988)), it can also be used to quantify akinesia (i.e. time to initiate a movement) also under conditions that are not characterized by increased muscle tone (i.e. rigidity) as in the cataleptic/catatonic state. Mice were gently placed on a table and forepaws were placed alternatively on blocks of increasing heights (1.5, 3 and 6 cm). The time (in seconds) that each paw spent on the block (i.e. the immobility time) was recorded (cut-off time of 20 s). Performance was expressed as total time spent on the different blocks.

### Drag test

Modification of the ‘wheelbarrow test’ (Schallert et al., 1979), this test measures the ability of the animal to balance its body posture with the forelimbs in response to an externally imposed dynamic stimulus (backward dragging) (Marti et al., 2005). It gives information regarding the time to initiate and execute (bradykinesia) a movement. Animals were gently lifted from the tail leaving the forepaws on the table, and then dragged backwards at a constant speed (about 20 cm/s) for a fixed distance (100 cm). The number of steps made by each paw was recorded. Five to seven determinations were collected for each animal.

### Rotarod test

The fixed-speed rotarod test (Rozas et al., 1997) measures different motor parameters such as motor coordination, gait ability, balance, muscle tone and motivation to run. Mice were tested over a wide range of increasing speeds (0–55 rpm; 5 rpm steps, increased every 180 s) on a rotating rod (diameter of the cylinder 8 cm) (Marti et al., 2004, Viaro et al., 2008) and the total time spent on the rod was recorded.

### Spontaneous motor activity

The open field test was used to measure spontaneous locomotor activity in 15-month-old mice. The ANY-maze video tracking system was used (Ugo Basile, application version 4.52c Beta) as previously described (Rizzi et al., 2008). Briefly, mice were placed in a square plastic cage (40 × 40 cm), one mouse per cage, and ambulatory behavior (horizontal activity) was monitored for 60 min with a camera. Four mice were monitored simultaneously each experiment. Total distance traveled (m) and immobility time (sec) were recorded.

### LRRK2 kinase inhibitor administration

Twelve-month-old mice were administrated i.p. with the LRRK2 kinase inhibitor H-1152 (Nichols et al., 2009, Sasaki et al., 2002, Tamura et al., 2005) at two different dose levels (0.1 and 1 mg/kg), or with the LRRK2 kinase inhibitor Nov-LRRK2-11 (Herzig et al., 2011, Troxler et al., 2013), at two different dose levels (1 and 10 mg/kg) for the indicated time. H-1152 was dissolved in 0.9% saline solution whereas Nov-LRRK2-11 in 3% DMSO/3% Tween 80.

### Cell culture and treatments

NIH3T3 cells were cultured in Dulbecco's Modified Eagle's medium (Life technologies) supplemented with 10% fetal bovine serum (Life technologies), penicillin and streptomycin (Life technologies) and maintained at 37 °C in a 5% CO_2_ controlled atmosphere. H-1152 (SiChem) and Nov-LRRK2-11 (Novartis) were dissolved in 0.9% saline solution and in 3% DMSO/3% Tween 80/0.9% saline, respectively. Inhibitors were used at the indicated concentrations, and equivalent volumes of saline solution were used as control. Inhibitors were added to the culture medium for 90 min before cell lysis.

### Cells and tissue lysis

NIH3T3 cells, as well as striatum and cortex obtained from brain dissection were homogenized and solubilized in lysis buffer (20 mM Tris–HCl pH 7.5, 150 mM NaCl, 1 mM EDTA, 2.5 mM sodium pyrophosphate, 1 mM β-glycerophosphate, 1 mM Na_3_VO_4_) supplemented with 1% Triton X-100 (Sigma Aldrich) and protease inhibitor cocktail (Roche), then cleared at 14,000 *g* at 4 °C for 30 min. Protein concentrations were determined using the bicinchoninic acid assay (BCA) as manufacturer's instructions (Thermo Scientific).

### Western blotting

Proteins were separated by electrophoresis into pre-casted 4–20% SDS-PAGE gels (Biorad) and subsequently transferred onto Immobilon-P membrane (Millipore). Membranes were first incubated 1 h at RT with rabbit anti-LRRK2 phospho Ser935 (1:300, Abcam, RabMAbs cat#ab133450), rabbit anti-LRRK2 UDD3 (1:1000, Abcam, RabMAbs cat# ab133518) and mouse anti-GADPH (1:4000, Millipore), then with HRP-conjugated secondary antibodies (Sigma) for 1 h at room temperature and then incubated with enhanced chemiluminescent (ECL) western blot substrate (Thermo Scientific).

### In vivo PK study

In order to gain insights on the brain penetration of Nov-LRRK2-11, a screening cassette approach was used, as previously described (Troxler et al., 2013). Adult male C57Bl/6 mice (20–30 g, Iffa-Credo, France) were orally administered (by gavage) with Nov-LRRK2-11 (suspended in carboxymethylcellulose 0.5 % w/v in water with Tween 80 at 0.5% v/v) at a dose of 3 mg/kg p.o.. Volume of oral administration was 10 mL/kg body weight.

After drug cassette administration, blood (~ 50 μL in EDTA) was collected at different time points (15 min, 30 min, 1, 2, 4, 8 and 24 h post-dose, n = 3 mice per time-point,) either by puncture of the sublingual vein (~ 70 μL/mouse, under light anesthesia) or by puncture from the vena cava at sacrifice (~ 300 μL/mouse). Moreover, at sacrifice (at 15 min, 1, 4, and 24 h post-dose), brains were removed, weighted and immediately frozen on dry ice. Blood and brain samples were stored at − 20 °C until analysis. Samples were analyzed for Nov-LRRK2-11 content with LC–MS/MS methodologies.

### Data presentation and statistical analysis

**Fig. 1:**
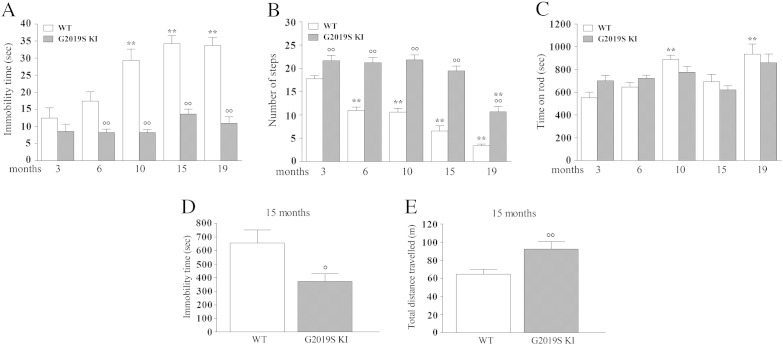
LRRK2 G2019S knock-in (G2019S KI) mice showed greater motor performances than wild-type littermates (WT). Motor performance was evaluated in two cohorts of mice following a longitudinal phenotyping approach. Mice were subjected to a set of tests, namely the bar (A), drag (B) and rotarod (C) tests, from 3 up to 19 months of age. In addition, an open field test was performed in 15-month old animals (D–E). Motor activity was expressed as immobility time (sec; A, D), number of steps (B), time on rod (sec; C), and total distance traveled (m; E). Data are means ± SEM of 9–10 (3–15 months) or 6 (19 months) mice per group, and were analyzed using one-way RM ANOVA followed by the Newman–Keuls test for multiple comparisons. Differences between genotypes at the single time-point levels were evaluated using the Student *t*-test, two tailed for unpaired data. *P < 0.05, **P < 0.01 different from 3-month-old mice of the same genotype. °P < 0.05,°°P < 0.01 different from age-matched littermates.

**Fig. 2:**
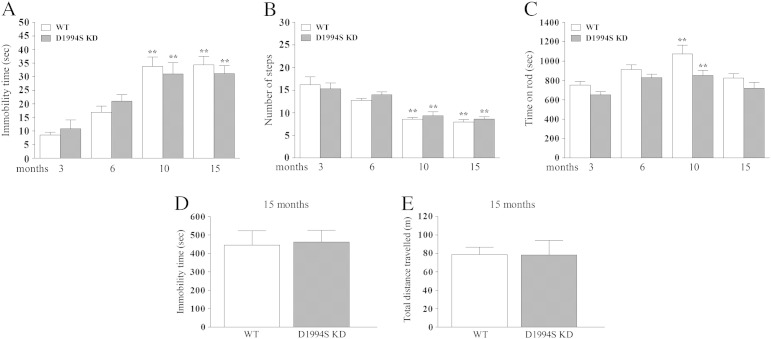
Mice carrying a LRRK2 mutation (D1994S) that silences kinase activity (kinase-dead; D1994S KD) showed similar motor phenotype of wild-type littermates (WT). Motor performance was evaluated in two cohorts of mice following a longitudinal phenotyping approach. Mice were subjected to a set of tests, namely the bar (A), drag (B) and rotarod (C) tests, starting at 3 months up to 19 months of age. In addition, an open field test was performed in 15-month-old animals (D–E). Motor activity was expressed as immobility time (sec; A, D), number of steps (B), time on rod (sec; C), and total distance traveled (m; E). Data are means ± SEM of 10 mice per group, and were analyzed using one-way RM ANOVA followed by the Newman–Keuls test for multiple comparisons. Differences between genotypes at the single time-point levels were evaluated using the Student *t*-test, two-tailed for unpaired data. **P < 0.01 different from 3-month-old mice of the same genotype.

Data are expressed as absolute values and are mean ± SEM (standard error of the mean) of n mice. To assess the significance of behavioral changes over the 19-month longitudinal study (Fig. 1, Fig. 2) a linear mixed-model repeated measures analysis using the REPEATED statement was used, followed by the Bonferroni test (PROC MIXED, SAS, version 9.2, SAS Institute Inc, Cary, NC, USA). Genotype was set as discrete variable, weight as continuous variable. This allowed to verify whether changes in weight could account for changes in behavioral performances. Statistical analysis of drug effect was performed by one-way repeated measure (RM) analysis of variance (ANOVA) followed by the Newman–Keuls test for multiple comparisons, or by two-way ANOVA followed by Bonferroni test for multiple comparisons (Table 1). Instead, two groups of data were compared with Student's *t*-test, two tailed for unpaired data. p-values < 0.05 were considered to be statistically significant.

**Table 1:** G2019S LRRK2 knock-in (KI) mice weighed less than wild-type littermates (WT) throughout the study. No difference was observed between D1994S LRRK2 kinase-dead (D1994S KD) and respective WT littermates. Data are expressed in grams and are means ± SEM of the number of mice indicated in parentheses. *P < 0.05, **P < 0.01 different from WT (2-way ANOVA followed by the Bonferroni post hoc test).

	Age (months)				
Genotype	3	6	9	15	19
G2019S KI (g)	23.8 ± 0.9** (11)	27.5 ± 0.4** (11)	30.4 ± 0.4** (11)	30.9 ± 0.4** (11)	31.8 ± 0.4* (6)
WT (g)	28.8 ± 0.8 (9)	31.5 ± 0.8 (9)	34.7 ± 0.8 (9)	35.3 ± 1.2 (9)	36.2 ± 1.4 (6)
D1994S KD (g)	25.6 ± 0.4 (10)	nt	32.0 ± 0.6 (10)	33.1 ± 0.7 (10)	nt
WT (g)	26.0 ± 0.4 (9)	nt	33.4 ± 0.7 (9)	34.6 ± 0.7 (9)	nt

nt = not tested.

## Results

### G2019S KI mice showed greater motor performance than WT mice

To investigate whether the kinase-enhancing G2019S point-mutation in murine LRRK2 affects motor performance, two cohorts of G2019S KI mice (n = 19) and age-matched WT littermates (WT; n = 12) were enrolled in a longitudinal study in which motor activity was measured using the bar, drag and rotarod tests from 3 through 19 months of age (Fig. 1).

G2019S KI mice had throughout the study a lower body weight than WT (Table 1). The difference was 14% on average.

In the bar test (Fig. 1A), a significant effect of genotype (F_1,75_ = 45.52, p < 0.0001), time (F_4,75_ = 4.47, p = 0.0027) and their interaction (F_4,75_ = 6.40, p = 0.0002) was found. The influence of weight was found not to be significant (F_1,75_ = 3.50, p = 0.07). Immobility time of G2019S KI mice in the bar test (8.5 ± 2.1 s) was not different from that of WT (12.4 ± 2.9 s) at 3 months (Fig. 1A). Immobility time increased along with aging in WT mice reaching a maximum of 33.7 ± 2.3 s at 19 months. Conversely, G2019S KI mice did not become akinetic with aging, showing similar performances across the study. The difference between genotypes was evident starting at 6 months (~ 2-fold), and attained stable values (~ 3-fold) from 10 months onward. In the drag test (Fig. 1B), a significant effect of genotype (F_1,75_ = 91.11, p < 0.0001), time (F_4,75_ = 26.78, p = 0.0027) and their interaction (F_4,75_ = 6.11, p = 0.0003) was found. As in the bar test, no significant influence of weight was observed (F_1,75_ = 1.54, p = 0.22). G2019S KI mice showed a significant 23% greater stepping activity than WT at 3 months (21.7 ± 1.1 vs 17.7 ± 0.7 steps, respectively). This difference became 2–3-fold larger in older animals, since stepping activity of WT mice progressively worsened over time, reaching 3.4 ± 0.3 steps at 19 months, whereas that of G2019S KI mice remained stable up to 15 months, showing a significant decline only at 19 months (10.7 ± 1.1 steps). Different from the bar and drag test, no significant effect of genotype was found in the rotarod test (F_1,75_ = 0.01, p = 0.92), but a significant effect of time (F_4,75_ = 8.24, p < 0.0001) and genotype × time interaction (F_4,75_ = 3.81, p = 0.0071). Also in this test, the influence of weight was not significant (F_1,75_ = 0.03, p = 0.86). Mild improvement of rotarod performance in WT mice was observed along with aging (10 and 19 months), whereas that of G2019S KI mice remained stable throughout the study (Fig. 1C). The open field test was performed in 15-month-old animals. G2019S KI mice showed 42% shorter immobility time (t = 2.53, df = 8, p = 0.036; Fig. 1D) and 43% longer distance traveled (t = 4.15, df = 8, p = 0.003; Fig. 1E) compared to WT.

To confirm the hyperkinetic phenotype of G2019S KI mice, the bar, drag, rotarod and open field tests were repeated in age-matched separate cohorts of 3, 10, 14 and 18-month-old mice, not involved in the longitudinal study (Fig. S1). These experiments substantially confirmed that G2019S KI mice were hyperactive, with significant differences with WT emerging already at 3 months in the bar and drag tests, and at 10 months in the open field. As in the longitudinal study, no differences in rotarod performance were observed between age-matched cohorts of WT and G2019S KI mice.

### D1994S KD mice behaved similar to WT in motor tests

Since experiments in G2019S KI mice suggested that enhancement of kinase activity is associated with greater motor performance, we investigated if kinase activity silencing mutation might cause a differential effect. To this purpose, we used mice bearing the kinase-inactivating point mutation D1994S (D1994S KD) and age-matched WT littermates.

No difference in weight was observed between D1994S KD and WT mice throughout the study (Table 1). Statistical analysis of bar test values revealed no significant effect of genotype (F_1,67_ = 0.1, p = 0.74), a significant effect of time (F_3,67_ = 31.23, p < 0.001) but not a genotype × time interaction (F_3,67_ = 1.17, p = 0.31). Likewise, in the drag test, a significant effect of time (F_3,67_ = 42.21, p < 0.001), but not of genotype (F_1,67_ = 0.48, p = 0.49) or genotype × time interaction (F_3,67_ = 0.56, p = 0.64) was found. Only in the rotarod test, a significant effect of genotype (F_1,67_ = 16.11, p = 0.0002) and time (F_3,67_ = 10.79, p < 0.0001) but not their interaction (F_3,67_ = 0.63, p = 0.62) was found. Overall, basal activity in the bar (Fig. 2A), drag (Fig. 2B) and rotarod (Fig. 2C) test was similar between D1994S KD mice and their WT at any age analyzed. As expected, WT but as well D1994S KD mice showed a significant worsening of motor activity in the bar (increase of immobility time) and drag (reduction of stepping activity) tests at 10 and 15 months. Transient improvement of rotarod performance was observed in WT and D1994S KD mice at 10 months. Consistently, no difference in exploratory behavior was observed between genotypes in the open field at 15 months (immobility time t = 0.21, df = 7, p = 0.84; distance traveled t = 0.03, df = 7, p = 0.97) Figs. 2D–E).

WT mice obtained from both colonies (G2019S KI and D1994S KD) showed substantially similar performances throughout the study (Tab. S1), with the exception of rotarod performance which was significantly lower at 3 and 6 months in WT littermates of G2019S KI mice.

### The LRRK2 inhibitor H-1152 reversed the motor phenotype of G2019S KI mice

**Fig. 3:**
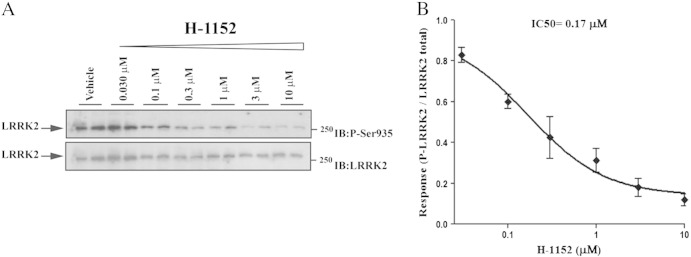
The LRRK2 kinase inhibitor H-1152 attenuated endogenous LRRK2 phosphorylation at Ser935 in cells. Blots from lysates of NIH3T3 cells (A) exposed to vehicle or increasing concentrations of H-1152 (0.03–10 μM) for 90 min (B). Data are means ± SEM of 2 experiments performed in duplicate.

Since results obtained with G2019S KI and D1994S KD mice suggest that the greater motor performance associated with the G2019S mutation is dependent on kinase activity, we next asked whether LRRK2 kinase inhibitors acutely administered to G2019S KI mice were effective at returning the hyperkinetic phenotypes to WT levels. We first used H-1152, a ROCK (Rho kinase) inhibitor (Nichols et al., 2009, Sasaki et al., 2002, Tamura et al., 2005) which has been previously shown to display high potency against LRRK2 (Gilsbach et al., 2012, Nichols et al., 2009). We initially confirmed that H-1152 was effective at inhibiting endogenous LRRK2 in NIH3T3 mouse fibroblasts, using de-phosphorylation of Ser935 as readout of LRRK2 kinase activity, as previously described (Dzamko et al., 2010). As shown in Fig. 3, H-1152 induced Ser935 de-phosphorylation in a concentration-dependent manner, with apparent IC_50_ of 170 nM.

**Fig. 4:**
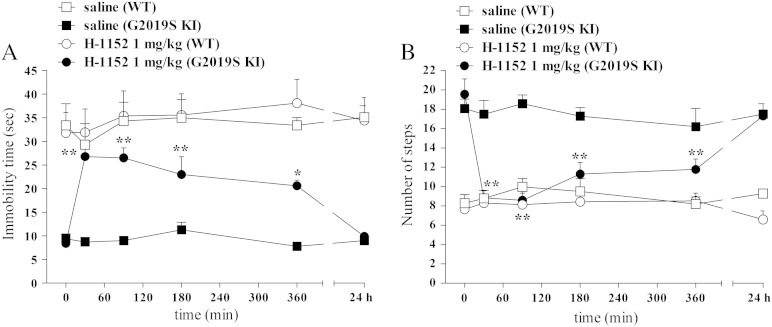
Time-course of the motor effects of the LRRK2 kinase inhibitor H-1152 in 12-month-old G2019S KI mice and wild-type littermates (WT). H-1152 (1 mg/kg; i.p.) or saline were administered to 12-month-old mice, and motor activity assessed using the bar (A) and drag (B) tests, before (time 0; basal values) and after (30, 90, 180, 360 min, 24 h) drug administration. Data are means ± SEM of 6 mice per group, and were analyzed using one-way RM ANOVA followed by the Newman–Keuls test for multiple comparisons. *P < 0.05, **P < 0.01 different from basal values.

**Fig. 5:**
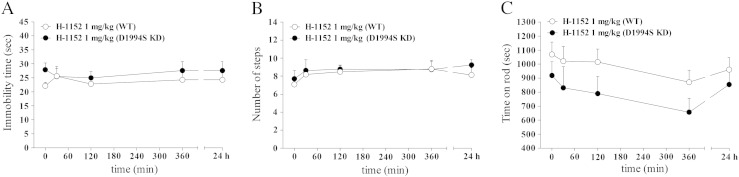
The LRRK2 kinase inhibitor H-1152 did not affect motor activity in mice carrying a LRRK2 mutation (D1994S) that silences kinase activity (kinase dead; D1994S KD). Time-course of the motor effects of H-1152 (1 mg/kg, i.p.) in 12-month-old D1994S KD mice and wild-type littermates (WT), assessed using the bar (A), drag (B) and rotarod (C) tests, before (time 0; basal values) and after (30, 120, 360 min, 24 h) drug administration. Data are means ± SEM of 6 mice per group, and were analyzed using one-way RM ANOVA followed by the Newman–Keuls test for multiple comparisons.

We next assessed H-1152 in vivo. In 6-month old mice, H-1152 was ineffective at 0.1 mg/kg, but increased immobility time (treatment F_2,21_ = 5.37, p = 0.013; time F_1,21_ = 0.72, p = 0.41; time × treatment interaction F_2,21_ = 10.87, p < 0.0001; Fig. S2A) and reduced stepping activity (treatment F_2,21_ = 4.69, p = 0.021; time F_1,21_ = 22.58, p < 0.0001; time × treatment interaction F_2,21_ = 5.93, p < 0.009; Fig. S2B) of G2019S KI mice to the levels of WT mice at 1 mg/kg. The same dose of H-1152 did not affect rotarod performance (treatment F_2,21_ = 0.06, p = 0.94; time F_1,21_ = 1.79, p = 0.19; time × treatment interaction F_2,21_ = 0.02, p = 0.98; Fig. S2C). The time-course of the response to H-1152 (1 mg/kg) was next studied in 12-month old mice (Fig. 4). Saline-treated WT and G2019S KI mice showed stable responses in the bar (Fig. 4A) and drag (Fig. 4B) tests across the 24-h observation period. Administration of H-1152 induced a rapid (maximal within 30 min) and prolonged (up to 6 h) increase of immobility time (treatment F_1,8_ = 59.32, p < 0.0001; time F_5,8_ = 14.25, p < 0.0001; time × treatment interaction F_5,40_ = 14.17, p < 0.0001; Fig. 4A) and reduction of stepping activity (treatment F_1,8_ = 14.64; p = 0.005; time F_5,8_ = 14.12, p < 0.0001; time × treatment interaction F_5,40_ = 13.61, p < 0.0001; Fig. 4B) in G2019S KI mice, being ineffective in WT mice (bar test: treatment F_1,8_ = 0.39, p = 0.84; time F_5,40_ = 2.77, p = 0.03; time × treatment interaction F_5,40_ = 0.92, p = 0.47; drag test: treatment F_1,8_ = 0.76, p = 0.41; time F_5,40_ = 1.57, p = 0.19; time × treatment interaction F_5,40_ = 1.94, p = 0.11 Fig. 4A). No residual effect of H-1152 was detected 24 h after administration. Rotarod performance was not significantly affected by H-1152 (G2019S KI: treatment F_1,8_ = 0.21, p = 0.65; time F_5,40_ = 6.27, p < 0.0001; time × treatment interaction F_5,40_ = 0.81, p = 0.54; WT: treatment F_1,8_ = 2.94, p = 0.12; time F_5,40_ = 10.85, p < 0.0001; time × treatment interaction F_5,40_ = 0.65, p = 0.64; Fig. S3). Indeed, performances worsened within 75 min after administration and remained stable afterwards in mice of both genotypes treated with saline or H-1152. Motor tests were repeated in 15-month old mice with substantially similar results (Fig. S4), although H-1152 (1 mg/kg) also mildly and transiently impaired rotarod performance in G2019S KI mice. To account for a certain compound specificity of LRRK2 activity inhibition we also treated D1994S KD mice and their wild-type controls (Figs. 5A-C). H-1152 (1 mg/kg) treatment did not affect motor activity in any genotypes (bar test: genotype F_1,8_ = 1.03, p = 0.34; time F_4,32_ = 0.34, p = 0.84; time × treatment interaction F_4,32_ = 0.53, p = 0.71; drag test: genotype F_1,8_ = 0.25, p = 0.63; time F_4,32_ = 2.28, p = 0.08; time × treatment interaction F_4,32_ = 0.34, p = 0.85; rotarod test: genotype F_1,8_ = 2.64, p = 0.14; time F_4,32_ = 3.98, p = 0.01; time × treatment interaction F_4,32_ = 0.79, p = 0.53), consistent with the lack of motor abnormalities in these animals.

**Fig. 6:**
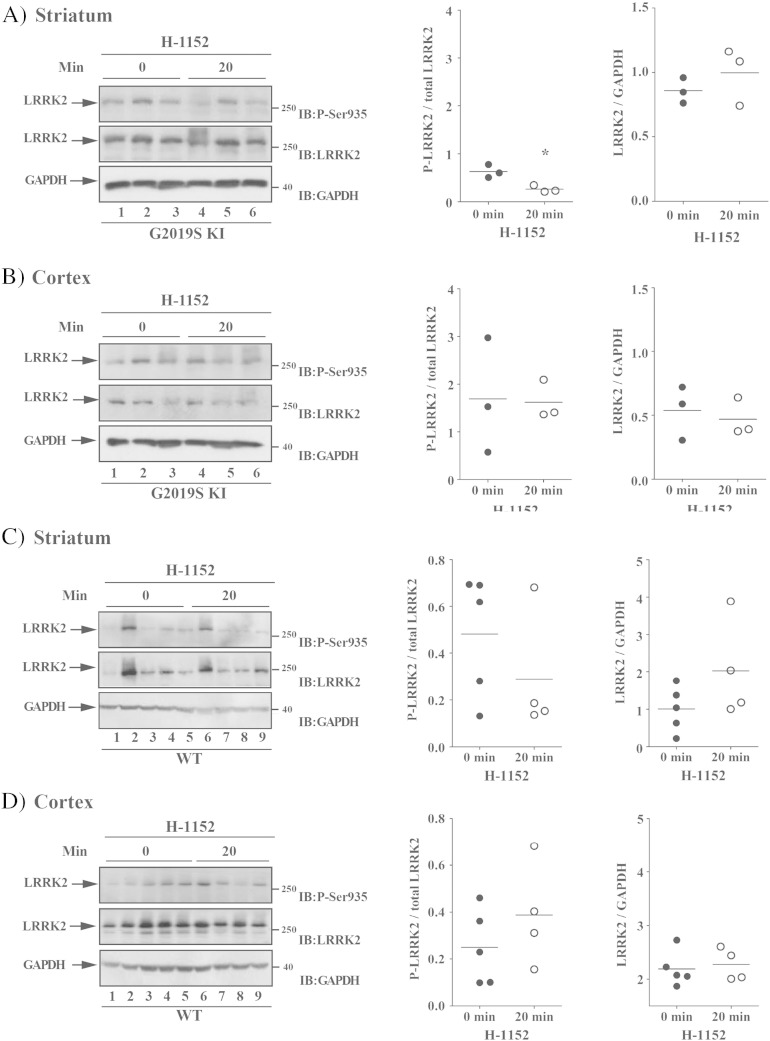
The LRRK2 kinase inhibitor H-1152 attenuated endogenous LRRK2 phosphorylation at Ser935 in striatum but not cerebral cortex of G2019S knock-in (G2019S KI) mice, being ineffective in wild-type littermates (WT). Three 12-month-old LRRK2 G2019S KI mice (A–B) were used as untreated controls, and three G2019S KI mice were administered with H-1152 (1 mg/kg, i.p.). In parallel, five WT KI mice (C–D) were used as controls, and four WT KI mice were treated with the same dose of H-1152 (1 mg/kg, i.p.). LRRK2 phosphorylation was measured ex-vivo in the striatum (A, C) and cerebral cortex (B, D), before (time 0; T0) and 20 min after H-1152 administration. Results are mean ± SEM of 3–5 mice per group, and were analyzed using the Student *t*-test, two tailed for unpaired data. To minimize experimental variability, each sample was loaded in duplicate and the numbers plotted represent the mean of the two technical replicates. *P < 0.05 different from basal values (T0).

Finally, we evaluated in vivo on-target engagement of H-1152 by measuring LRRK2 phosphorylation at Ser935 in ex vivo samples of the striatum and cerebral cortex obtained from 12-month old G2019S KI (Figs. 6A-B) and WT (Figs. 6C-D) mice. A decrease of LRRK2 phosphorylation was observed in striatum (p = 0.015, t = 4.05, df = 4), but not cerebral cortex (p = 0.095, t = 0.98, df = 4), at 20 min after administration of 1 mg/kg H-1152 but not later time-points (i.e. 90 and 360 min; Figs. S5A-B). Contrary to G2019S KI mice, no effect of H-1152 on LRRK2 phosphorylation was detected in striatum (p = 0.30, t = 1.11, df = 7) or cerebral cortex (p = 0.31, t = 1.08, df = 7) at 20 min after administration in WT mice (Figs. 6B-D)

### Nov-LRRK2-11 confirmed that the hyperactivity of G2019S KI mice is kinase-dependent

**Fig. 7:**
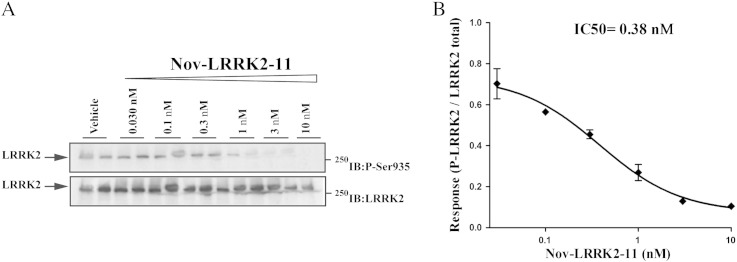
The LRRK2 kinase inhibitor Nov-LRRK2-11 attenuated endogenous LRRK2 phosphorylation at Ser935 in cells. Blots from lysates of NIH3T3 cells (A) exposed to vehicle or increasing concentrations of Nov-LRRK2-11 (0.03–10 nM) for 90 min (B). Data are means ± SEM of 2 experiments performed in duplicate.

**Fig. 8:**
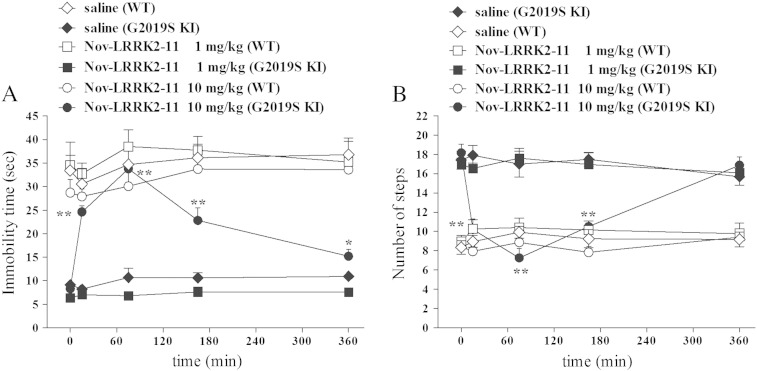
The LRRK2 kinase inhibitor Nov-LRRK2-11 reversed motor phenotype in G2019S knock-in (G2019S KI) mice being ineffective in wild-type littermates (WT). Time-course of the motor effects of Nov-LRRK2-11 (1 and 10 mg/kg, i.p.), in comparison with saline, in 12-month-old G2019S KI and WT mice. Motor activity was assessed using the bar (A) and drag (B) tests, before (time 0; basal values) and after (15, 75, 165, 360 min) drug administration. Data are means ± SEM of 6–8 mice per group, and were analyzed using one-way RM ANOVA followed by the Newman–Keuls test for multiple comparisons. *P < 0.05, **P < 0.01 different from basal values.

To confirm that the results obtained with H-1152 are due to LRRK2 inhibition and not other off-target kinases, we employed a second small molecule ATP analog inhibitor, Nov-LRRK2-11, which has been recently shown to be brain penetrant and reasonably selective (Troxler et al., 2013). We first tested Nov-LRRK2-11 in vitro for its ability to inhibit LRRK2 Ser935 phosphorylation. Nov-LRRK2-11 resulted in being very potent at reducing LRRK2 phosphorylation in NIH3T3 cells, with IC_50_ of 0.38 nM (Fig. 7). Next, we assessed the compound in vivo (Fig. 8). Nov-LRRK2-11 (1 and 10 mg/kg, i.p.) did not induce any obvious behavioral change on immobility time and step number in WT mice (bar test: treatment F_2,13_ = 39.83, p < 0.001; time F_4,52_ = 2.33, p = 0.07; time × treatment interaction F_8,52_ = 0.75, p = 0.65; drag test: treatment F_2,13_ = 1.05, p = 0.37; time F_4,52_ = 2.34, p = 0.07; time × treatment interaction F_8,52_ = 0.95, p = 0.48; Figs. 8A-B). However, Nov-LRRK2-11 acute treatment phenocopied the motor inhibiting effects of H-1152 in G2019S KI mice (bar test: treatment F_2,13_ = 60.05, p < 0.0001; time F_4,60_ = 29.15, p < 0.0001; time × treatment interaction F_8,60_ = 0.29.15, p < 0.0001; drag test: treatment F_2,15_ = 12.65, p = 0.001; time F_4,60_ = 18.51, p < 0.0001; time × treatment interaction F_8,60_ = 15.55, p < 0.0001; Figs. 8A-B). Nov-LRRK2-11 was ineffective at 1 mg/kg, and induced a rapid (significant at 20 min, maximal at 75 min) increase in immobility time (Fig. 8A) and reduction of stepping activity (Fig. 8B) at 10 mg/kg. These effects were shorter lasting than those of H-1152, since stepping activity was normalized and immobility time only mildly elevated at 360 min after administration. Nov-LRRK2-11 caused a delayed reduction of rotarod performance in WT mice at 1 and 10 mg/kg, the latter dose inducing a more rapid effect (rotarod test: treatment F_2,13_ = 0.78, p = 0.47; time F_4,52_ = 12.96, p < 0.0001; time × treatment interaction F_8,52_ = 3.52, p = 0.002; 175 min; Fig. S6). In G2019S KI mice, the lower dose induced a response that was superimposable to that observed in WT, albeit more rapid in onset (within 15 min) (rotarod test: treatment F_2,20_ = 0.94, p = 0.40; time F_4,80_ = 18.37, p < 0.0001; time × treatment interaction F_8,80_ = 3.29, p = 0.003). Behavioral data were in line with pharmacokinetic data. In fact, following an oral dose of 3 mg/kg Nov-LRRK2-11, brain and blood concentrations were maximal at 1 h, and only minimally detected at 4 h; at 24 h, compound levels were below detection (Fig. S7).

**Fig. 9:**
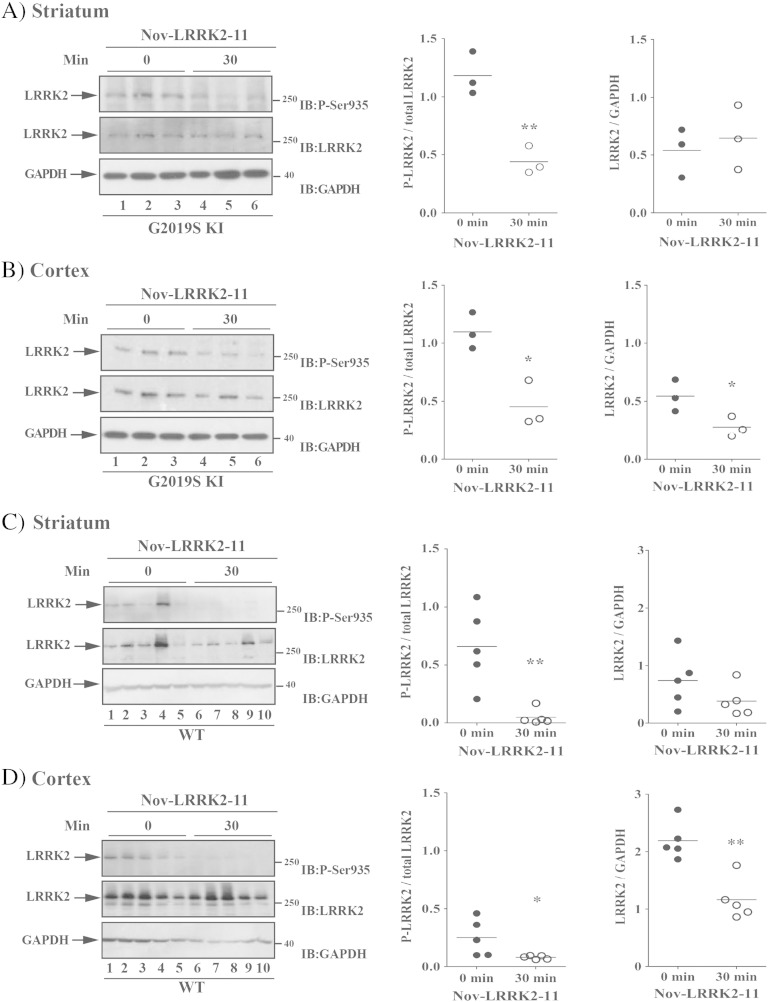
The LRRK2 kinase inhibitor Nov-LRRK2-11 attenuated endogenous LRRK2 phosphorylation at Ser935 in the striatum and cortex of both G2019S knock-in (G2019S KI) mice and wild-type littermates (WT). Three 12-month-old G2019S KI mice (A–B) were used as untreated controls, and three G2019S KI mice were administered with Nov-LRRK2-11 (10 mg/kg, i.p.). In parallel, five WT KI mice (C–D) were used as controls, and five WT KI mice were treated with the same dose of Nov-LRRK2-11 (10 mg/kg, i.p.). LRRK2 phosphorylation was measured ex vivo in the striatum (A, C) and cerebral cortex (B, D), before (time 0; T0) and 30 min after Nov-LRRK2-11 administration. Results are mean ± SEM of 3–5 mice per group, and were analyzed using the Student *t*-test, two tailed for unpaired data. *P < 0.05, **P < 0.01 different from basal values (T0).

To confirm in vivo LRRK2 targeting, we measured LRRK2 phosphorylation at Ser935 30 min after Nov-LRRK2-11 (10 mg/kg) administration in 12-month old G2019S KI and WT animals (Fig. 9). Nov-LRRK2-11 markedly reduced LRRK2 phosphorylation in the striatum and cerebral cortex of G2019S KI mice (by 75% and 50%, respectively, n = 3 animals each; striatum p = 0.008, t = 4.78, df = 4; cortex p = 0.011, t = 4.41, df = 4; Figs. 9A-B) as well as WT mice (by 60% and 80%, respectively, n = 5 animals each, striatum p = 0.004, t = 3.95, df = 8; cortex p = 0.044, t = 2.37, df = 8; Figs. 9C-D). Interestingly, in the cerebral cortex but not striatum, pharmacological blockade of LRRK2 kinase activity reduced protein LRRK2 levels (Figs. 9B and D).

Finally, pSer935 LRRK2 and endogenous LRRK2 protein levels were monitored in 12-month-old G2019S KI and KD mice in comparison with their WT controls (Fig. S8). pSer935 LRRK2 levels in striatum and cortex, as well as LRRK2 protein levels in striatum were similar across genotypes. Likewise, similar levels of LRRK2 were found in the cortex of G2019S KI and WT mice. D19994S KD levels were also in the same range of G2019S KI mice, although lower than those found in their littermates.

## Discussion

Previous studies have attempted to replicate a parkinsonian-like phenotype in rodents by overexpressing pathogenic G2019S LRRK2 mutation. These studies differ in many ways, such as the technology used (BAC or cDNA trangenesis, viral vectors), the levels of transgene expression and its neuronal localization, the mouse strain and, not last, the motor tests used. Nonetheless, these studies failed in showing a detrimental effect of G2019S on motor functions, unless high levels of transgene expression are artificially attained in *substantia nigra* neurons via the CMV/PDGF promoter, leading to 30–50% neuronal loss (Chen et al., 2012). Consistent with this view, no motor change was observed in another study on these mice (although bred on a different background), where a lower level of transgene expression in DA neurons and, consequently, a lower degree of *substantia nigra* neurodegeneration was achieved (18%) (Ramonet et al., 2011).

To extend previous studies, here we provide the results of the first longitudinal phenotyping study in G2019S KI mice, showing that expression of mouse *LRRK2* gene carrying the G2019S mutation confers a hyperkinetic phenotype, that is resistant to age-related motor decline. The robust and long lasting hyperkinetic phenotype described was substantially confirmed by a transversal motor analysis of different age-matched cohorts of G2019S KI and WT mice.

Two lines of evidence seem to confirm that enhancement of kinase activity, which is a consequence of the G2019S mutation (Greggio et al., 2006, Jaleel et al., 2007, West et al., 2005), increases motor performance: i) the motor function of mice carrying a kinase-silencing mutation (D1994S) showed normal age-related motor worsening superimposable to that of WT littermates and ii) ATP-competitive kinase inhibitors reversed the hyperkinetic phenotype selectively in G2019S KI mice. Further evidence that enhancement of LRRK2 kinase activity might be responsible for the observed motor phenotype in G2019S KI mice comes from KI mice carrying the R1441C mutation in the ROC domain (Xiong et al., 2012). In fact, the R1441C mutation induces a milder increase of kinase activity with respect to the G2019S mutation (West et al., 2005), or no increase at all (Jaleel et al., 2007), and motor activity of R1441C KI mice in the open field and rotarod appears to be unchanged up to 24 months of age (Tong et al., 2009).

The motor phenotype described in the present study clearly differs from that reported in G2019S overexpressing mice, although transient and test-related motor facilitation has been observed in those mice. For instance, BAC mice overexpressing human G2019S LRRK2 showed faster walking speed (and anxiety-like behavior) in the open field (Melrose et al., 2010) whereas human G2019S overexpressors under the Thy1 (Herzig et al., 2012) or the TetO CaMKII (Lin et al., 2009) promoters showed transient improvement in rotarod performance (Herzig et al., 2012) or increased exploratory behavior (Lin et al., 2009). In addition, rats temporarily, but not constitutively, overexpressing human G2019S LRRK2 showed increased exploratory behavior in the open field at 18 months (Zhou et al., 2011).

The influence of G2019S LRRK2 on motor function was evident in tests specific for akinesia/bradykinesia (bar and drag tests), and spontaneous exploratory behavior (open field), but not in a test for exercise-driven motor activity (rotarod). This might suggest an influence of G2019S LRRK2 on specific motor parameters. Indeed, stepping activity involves striatal sensory–motor function (Kirik et al., 1998) whereas the rotarod test integrates both motor and non-motor (e.g. motivation to run) functions, and likely involves multiple brain areas (e.g. basal ganglia and cerebellum), where LRRK2 is expressed to a different degree (Higashi et al., 2007, Melrose et al., 2007). However, both in the bar and drag test, the main effect of G2019S was to preserve motor function from aging, suggesting that, more in general, G2019S mutation might confer a phenotype which is more resistant (or less sensitive) to the age-related motor decline.

The possibility that this hyperkinetic phenotype is associated with changes of neurotransmitter release should be considered. Indeed, both LRRK2 silencing (Piccoli et al., 2011) or expression of the G2019S mutation (Migheli et al., 2013) has been reported to facilitate exocytosis, consistent with the finding that too much or too little kinase activity has a negative impact on vesicle trafficking (Matta et al., 2012). Consistently, pharmacological inhibition of LRRK2 activity impairs vesicle endocytosis and neurotransmitter release (Cirnaru et al., 2014). Moreover, enhancement of the stimulus-induced DA release has been detected in PC12 cells expressing the G2019S mutation (Migheli et al., 2013). Therefore, we might hypothesize that the hyperkinetic phenotype of G2019S KI mice is due to increased DA concentration at the synaptic cleft. Alternatively, an increased motor activity could result, even in the absence of an elevation of DA levels, from an increased expression of postsynaptic D1 receptors, as suggested by study in G2019S expressing cells (Migheli et al., 2013).

The motor phenotype of G2019S KI mice seems to be achieved through a gain-of-function process. Indeed, silencing kinase activity does not affect motor function. The lack of endogenous control over motor activity by endogenous LRRK2 is further supported by the absence of a clear motor phenotype in LRRK2 knockout mice (Herzig et al., 2011, Lin et al., 2009), although exploratory changes consistent with anxiety-like behavior, and (transient) facilitation of rotarod performance have also been reported in these mice (Hinkle et al., 2012). While our results disclose robust motor alterations in G2019S KI mice, KI mice expressing other pathological LRRK2 mutations as well as G2019S KI mice from other laboratories need to be assessed in these motor tests to strengthen the involvement of LRRK2 kinase activity in this paradigm.

Interestingly, mice carrying PD-linked mutations, such as α-synuclein overexpressed under the Thy1 promoter (Lam et al., 2011), or parkin and DJ-1 double knock-out mice (Hennis et al., 2014) were found to be hyperactive and have increased striatal DA levels in their pre-symptomatic phase, possibly indicating compensatory mechanisms preceding nigro-striatal DA system demise. Whether the hyperactive motor phenotype of G2019S KI mice might be considered as a result of pre-symptomatic compensatory changes is presently under investigation. To possibly support this view, asymptomatic human G2019S carriers have higher putaminal DA turnover rate (Sossi et al., 2010). The fact that we did not observe a reversal of motor hyperactivity into frank hypokinesia, as in α-synuclein overexpressors (Lam et al., 2011), up to 19 months, might indicate a longer and slower pre-symptomatic phase in G2019S KI mice, in line with the different ages at onset of the disease, i.e. juvenile for α-synuclein related PD, and late for G2019S related PD (Hardy et al., 2009). It will be interesting to investigate in the future the molecular basis underlying LRRK2 G2019S-related hyperactive motor performance. Indeed, the requirement of kinase activity in LRRK2 pathogenic effects is still unclear. For instance, there is also growing evidence that the LRRK2 levels are driving the neuropathology rather than the kinase activity (Herzig et al., 2011, Skibinski et al., 2014). Nevertheless, the role of LRRK2 on motor function and on neurotoxicity can be two independent mechanisms.

Another important finding of the present study is the demonstration that ATP-competitive LRRK2 kinase inhibitors reversed the motor phenotype in G2019S KI mice. Both H-1152 and Nov-LRRK2-11 were able to inhibit LRRK2 kinase activity and reduce LRRK2 phosphorylation at Ser935 in NIH3T3 cells. This confirms previous findings that LRRK2 kinase inhibition with H-1152 abolishes binding to 14-3-3 proteins, resulting in de-phosphorylation of LRRK2 at Ser910 and Ser935 (Dzamko et al., 2010). Based on these data, phosphorylation at Ser935 has been proposed as a readout of LRRK2 kinase activity (Dzamko et al., 2010) although this phosphorylation is not the direct consequence of autophosphorylation but is possibly controlled by a LRRK2-activated kinase/phosphatase and, therefore, does not always correlate with kinase activity (Dzamko et al., 2012).

In our hands, Nov-LRRK2-11 reversed the phenotype of G2019S KI mice and inhibited LRRK2 phosphorylation at 10 mg/kg, confirming ex vivo pulldown experiments showing that Nov-LRRK2-11 penetrates into the brain (Troxler et al., 2013). Behavioral data were in agreement with PK data after 3 mg/kg oral dose, and with the apparent terminal half-life of Nov-LRRK2-11 in blood after i.v. dosing of 1 mg/kg (0.4 h) (Herzig et al., 2011, Troxler et al., 2013).

Interestingly, the same behavioral effect was observed at ten-fold lower doses of H-1152, suggesting significant brain penetration also for H-1152, for which, however, no published pharmacokinetic are available. Further validation of this motor reversal phenotype with additional compounds inhibiting LRRK2 is of course desirable and necessary.

The present study shows a clear dissociation between motor changes and in vivo LRRK2 phosphorylation. Indeed, Nov-LRRK2-11 inhibited motor activity in G2019S KI mice causing only minimal effects (on rotarod performance) in WT mice, in face of a marked inhibition of LRRK2 phosphorylation in the striatum and cerebral cortex of both genotypes. Moreover, although H-1152 consistently inhibited motor activity and LRRK2 phosphorylation in G2019S KI but not WT mice, its motor effects far exceeded those on LRRK2 phosphorylation.

The most parsimonious way to explain this discrepancy is that LRRK2 de-phosphorylation at Ser935 is a marker for in vivo target engagement of LRRK2 kinase inhibitors, but does not follow their motor effects. Perhaps, other phosphorylation, or autophosphorylation (Sheng et al., 2012), site(s) on LRRK2 should be monitored. In fact, we should recall that both inhibitors target other kinases beyond LRRK2, which may act upstream and downstream of LRRK2. For instance, PKA has been shown to crosstalk with LRRK2 (Li et al., 2011, Muda et al., 2014, Parisiadou et al., 2014), whereas ROCK/MLCK are involved in actin cytoskeleton remodeling pathways, in which activity they could crosstalk with LRRK2.

On the other hand, given the parallel (albeit temporally-dissociated) inhibition of motor activity and striatal LRRK2 phosphorylation induced by H-1152, we might speculate that striatal LRRK2 is a key regulator of motor activity. In this case, if higher LRRK2 kinase activity is present in the striatum of G2019S KI mice compared to WT mice, as predicted, normalization of these levels by LRRK2 kinase inhibitors might represent the trigger of a cascade of events leading to sustained motor inhibition.

Moreover, the different patterns of LRRK2 de-phosphorylation of Nov-LRRK2-11 and H-1152 in G2019S KI and WT mice suggest a higher LRRK2 selectivity and/or brain exposure/free fraction of Nov-LRRK2-11. For further confirmation, additional information on earlier times-points (< 20 min) of Nov-LRRK2-11 effects as well as higher doses of H-1152 would be desirable. In addition, the finding that Nov-LRRK2-11 reduces LRRK2 levels in the cortex but not striatum indicates different properties of the LRRK2 system in these two areas.

### Concluding remarks

The present longitudinal phenotypic study provides genetic evidence that expression of the G2019S mutation under the endogenous promoter confers mice with better motor performances and preserves their age-related motor decline in tests specific for akinesia/bradykinesia. Enhancement of LRRK2 kinase activity likely underlies this phenotype since D1994S KD mice do not display motor abnormalities, and ATP-competitive LRRK2 inhibitors reversed motor phenotype in G2019S KI mice. This study challenges the idea that G2019S is detrimental for motor activity in rodents, suggesting that other factors might be involved in inducing a PD-like phenotype, such alpha-synuclein (for reviews see (Greggio et al., 2011, Taymans and Cookson, 2010) or parkin (Smith et al., 2005). The possibility that the hyperkinetic phenotype of G2019S KI mice might reflect a pre-symptomatic stage of PD needs also to be explored. Finally, but not less important, this study also describes for the first time a correlation between in vivo motor effects of LRRK2 inhibitors and their ability to de-phosphorylate LRRK2 at Ser935, suggesting the G2019S KI mice may represent a valuable in vivo model to screen for LRRK2 inhibitors.

## Appendix A Supplementary data

Table S1. Motor performances of WT mice used in the study. Figure S1. Motor performances in aged-matched cohorts of G2019S KI and WT mice. Figure S2. H-1152 in 6-month-old mice. Figure S3. Rotarod performance in 12-month-old mice treated with H-1152. Figure S4. H-1152 in 15-month-old mice. Figure S5. H-1152 on pSer935 LRRK2 at 90 and 360 min after administration. Figure S6. Rotarod performance in 12-month-old mice treated with Nov-LRRK2-11. Figure S7. Blood and brain levels of Nov-LRRK2-11. Figure S8. pSer935 LRRK2 and LRRK2 protein levelsin striatum and cerebral cortex.

## References

[bb0005] Barrett J.C. (2008). Genome-wide association defines more than 30 distinct susceptibility loci for Crohn's disease. Nat. Genet..

[bb0010] Chen C.Y. (2012). (G2019S) LRRK2 activates MKK4-JNK pathway and causes degeneration of SN dopaminergic neurons in a transgenic mouse model of PD. Cell Death Differ..

[bb0015] Choi H.G. (2012). Brain penetrant LRRK2 inhibitor. ACS Med. Chem. Lett..

[bb0020] Cirnaru M.D. (2014). LRRK2 kinase activity regulates synaptic vesicle trafficking and neurotransmitter release through modulation of LRRK2 macro-molecular complex. Front. Mol. Neurosci..

[bb0025] Cookson M.R. (2010). The role of leucine-rich repeat kinase 2 (LRRK2) in Parkinson's disease. Nat. Rev. Neurosci..

[bb0030] Dzamko N. (2010). Inhibition of LRRK2 kinase activity leads to dephosphorylation of Ser(910)/Ser(935), disruption of 14-3-3 binding and altered cytoplasmic localization. Biochem. J..

[bb0035] Dzamko N. (2012). The IkappaB kinase family phosphorylates the Parkinson's disease kinase LRRK2 at Ser935 and Ser910 during Toll-like receptor signaling. PLoS One.

[bb0045] Estrada A.A. (2012). Discovery of highly potent, selective, and brain-penetrable leucine-rich repeat kinase 2 (LRRK2) small molecule inhibitors. J. Med. Chem..

[bb0040] Estrada A.A. (2014). Discovery of highly potent, selective, and brain-penetrant aminopyrazole leucine-rich repeat kinase 2 (LRRK2) small molecule inhibitors. J. Med. Chem..

[bb0050] Gilsbach B.K. (2012). Roco kinase structures give insights into the mechanism of Parkinson disease-related leucine-rich-repeat kinase 2 mutations. Proc. Natl. Acad. Sci. U. S. A..

[bb0060] Greggio E. (2006). Kinase activity is required for the toxic effects of mutant LRRK2/dardarin. Neurobiol. Dis..

[bb0055] Greggio E. (2011). Leucine-rich repeat kinase 2 and alpha-synuclein: intersecting pathways in the pathogenesis of Parkinson's disease?. Mol. Neurodegener..

[bb0065] Hardy J. (2009). The genetics of Parkinson's syndromes: a critical review. Curr. Opin. Genet. Dev..

[bb0070] Hassin-Baer S. (2009). The leucine rich repeat kinase 2 (LRRK2) G2019S substitution mutation. Association with Parkinson disease, malignant melanoma and prevalence in ethnic groups in Israel. J. Neurol..

[bb0075] Healy D.G. (2008). Phenotype, genotype, and worldwide genetic penetrance of LRRK2-associated Parkinson's disease: a case–control study. Lancet Neurol..

[bb0080] Hennis M.R. (2014). Surprising behavioral and neurochemical enhancements in mice with combined mutations linked to Parkinson's disease. Neurobiol. Dis..

[bb0090] Herzig M.C. (2011). LRRK2 protein levels are determined by kinase function and are crucial for kidney and lung homeostasis in mice. Hum. Mol. Genet..

[bb0085] Herzig M.C. (2012). High LRRK2 levels fail to induce or exacerbate neuronal alpha-synucleinopathy in mouse brain. PLoS One.

[bb0095] Higashi S. (2007). Expression and localization of Parkinson's disease-associated leucine-rich repeat kinase 2 in the mouse brain. J. Neurochem..

[bb0100] Hinkle K.M. (2012). LRRK2 knockout mice have an intact dopaminergic system but display alterations in exploratory and motor co-ordination behaviors. Mol. Neurodegener..

[bb0105] Iaccarino C. (2007). Apoptotic mechanisms in mutant LRRK2-mediated cell death. Hum. Mol. Genet..

[bb0110] Jaleel M. (2007). LRRK2 phosphorylates moesin at threonine-558: characterization of how Parkinson's disease mutants affect kinase activity. Biochem. J..

[bb0115] Kirik D. (1998). Characterization of behavioral and neurodegenerative changes following partial lesions of the nigrostriatal dopamine system induced by intrastriatal 6-hydroxydopamine in the rat. Exp. Neurol..

[bb0120] Kumar V. (2013). Genome-wide association study signal at the 12q12 locus for Crohn's disease may represent associations with the MUC19 gene. Inflamm. Bowel Dis..

[bb0125] Kuschinsky K., Hornykiewicz O. (1972). Morphine catalepsy in the rat: relation to striatal dopamine metabolism. Eur. J. Pharmacol..

[bb0130] Lam H.A. (2011). Elevated tonic extracellular dopamine concentration and altered dopamine modulation of synaptic activity precede dopamine loss in the striatum of mice overexpressing human alpha-synuclein. J. Neurosci. Res..

[bb0135] Lee B.D. (2010). Inhibitors of leucine-rich repeat kinase-2 protect against models of Parkinson's disease. Nat. Med..

[bb0140] Li X. (2010). Enhanced striatal dopamine transmission and motor performance with LRRK2 overexpression in mice is eliminated by familial Parkinson's disease mutation G2019S. J. Neurosci..

[bb0145] Li X. (2011). Phosphorylation-dependent 14-3-3 binding to LRRK2 is impaired by common mutations of familial Parkinson's disease. PLoS One.

[bb0150] Lin X. (2009). Leucine-rich repeat kinase 2 regulates the progression of neuropathology induced by Parkinson's-disease-related mutant alpha-synuclein. Neuron.

[bb0155] Liu Z. (2011). Inhibitors of LRRK2 kinase attenuate neurodegeneration and Parkinson-like phenotypes in *Caenorhabditis elegans* and *Drosophila* Parkinson's disease models. Hum. Mol. Genet..

[bb0160] Marin I. (2006). The Parkinson disease gene LRRK2: evolutionary and structural insights. Mol. Biol. Evol..

[bb0170] Marti M. (2004). Blockade of nociceptin/orphanin FQ receptor signaling in rat substantia nigra pars reticulata stimulates nigrostriatal dopaminergic transmission and motor behavior. J. Neurosci..

[bb0165] Marti M. (2005). Blockade of nociceptin/orphanin FQ transmission attenuates symptoms and neurodegeneration associated with Parkinson's disease. J. Neurosci..

[bb0175] Marti M. (2007). The nociceptin/orphanin FQ receptor antagonist J-113397 and L-DOPA additively attenuate experimental parkinsonism through overinhibition of the nigrothalamic pathway. J. Neurosci..

[bb0180] Mata I.F. (2006). LRRK2 in Parkinson's disease: protein domains and functional insights. Trends Neurosci..

[bb0185] Matta S. (2012). LRRK2 controls an EndoA phosphorylation cycle in synaptic endocytosis. Neuron.

[bb0195] Melrose H.L. (2007). A comparative analysis of leucine-rich repeat kinase 2 (Lrrk2) expression in mouse brain and Lewy body disease. Neuroscience.

[bb0190] Melrose H.L. (2010). Impaired dopaminergic neurotransmission and microtubule-associated protein tau alterations in human LRRK2 transgenic mice. Neurobiol. Dis..

[bb0200] Migheli R. (2013). LRRK2 affects vesicle trafficking, neurotransmitter extracellular level and membrane receptor localization. PLoS One.

[bb0205] Muda K. (2014). Parkinson-related LRRK2 mutation R1441C/G/H impairs PKA phosphorylation of LRRK2 and disrupts its interaction with 14-3-3. Proc. Natl. Acad. Sci. U. S. A..

[bb0210] Nichols R.J. (2009). Substrate specificity and inhibitors of LRRK2, a protein kinase mutated in Parkinson's disease. Biochem. J..

[bb0215] Paisan-Ruiz C. (2004). Cloning of the gene containing mutations that cause PARK8-linked Parkinson's disease. Neuron.

[bb0220] Parisiadou L. (2014). LRRK2 regulates synaptogenesis and dopamine receptor activation through modulation of PKA activity. Nat. Neurosci..

[bb0225] Piccoli G. (2011). LRRK2 controls synaptic vesicle storage and mobilization within the recycling pool. J. Neurosci..

[bb0230] Ramonet D. (2011). Dopaminergic neuronal loss, reduced neurite complexity and autophagic abnormalities in transgenic mice expressing G2019S mutant LRRK2. PLoS One.

[bb0235] Rizzi A. (2008). Neuropeptide S is a stimulatory anxiolytic agent: a behavioural study in mice. Br. J. Pharmacol..

[bb0240] Rozas G. (1997). An automated rotarod method for quantitative drug-free evaluation of overall motor deficits in rat models of parkinsonism. Brain Res. Brain Res. Protocol..

[bb0245] Sanberg P.R. (1988). The catalepsy test: its ups and downs. Behav. Neurosci..

[bb0250] Sasaki Y. (2002). The novel and specific Rho-kinase inhibitor (S)-(+)-2-methyl-1-[(4-methyl-5-isoquinoline)sulfonyl]-homopiperazine as a probing molecule for Rho-kinase-involved pathway. Pharmacol. Ther..

[bb0255] Schallert T. (1979). Excessive bracing reactions and their control by atropine and L-DOPA in an animal analog of Parkinsonism. Exp. Neurol..

[bb0260] Sheng Z. (2012). Ser1292 autophosphorylation is an indicator of LRRK2 kinase activity and contributes to the cellular effects of PD mutations. Sci. Transl. Med..

[bb0265] Skibinski G. (2014). Mutant LRRK2 toxicity in neurons depends on LRRK2 levels and synuclein but not kinase activity or inclusion bodies. J. Neurosci..

[bb0270] Smith W.W. (2005). Leucine-rich repeat kinase 2 (LRRK2) interacts with parkin, and mutant LRRK2 induces neuronal degeneration. Proc. Natl. Acad. Sci. U. S. A..

[bb0275] Sossi V. (2010). Dopamine turnover increases in asymptomatic LRRK2 mutations carriers. Mov. Disord..

[bb0280] Tamura M. (2005). Development of specific Rho-kinase inhibitors and their clinical application. Biochim. Biophys. Acta.

[bb0285] Taymans J.M., Cookson M.R. (2010). Mechanisms in dominant parkinsonism: the toxic triangle of LRRK2, alpha-synuclein, and tau. Bioessays.

[bb0290] Tong Y. (2009). R1441C mutation in LRRK2 impairs dopaminergic neurotransmission in mice. Proc. Natl. Acad. Sci. U. S. A..

[bb0295] Troxler T. (2013). Discovery of novel indolinone-based, potent, selective and brain penetrant inhibitors of LRRK2. Bioorg. Med. Chem. Lett..

[bb0300] Viaro R. (2008). Nociceptin/orphanin FQ receptor blockade attenuates MPTP-induced parkinsonism. Neurobiol. Dis..

[bb0305] West A.B. (2005). Parkinson's disease-associated mutations in leucine-rich repeat kinase 2 augment kinase activity. Proc. Natl. Acad. Sci. U. S. A..

[bb0310] Xiong Y. (2012). LRRK2 GTPase dysfunction in the pathogenesis of Parkinson's disease. Biochem. Soc. Trans..

[bb0315] Yue Z., Lachenmayer M.L. (2011). Genetic LRRK2 models of Parkinson's disease: dissecting the pathogenic pathway and exploring clinical applications. Mov. Disord..

[bb0320] Zhang F.R. (2009). Genomewide association study of leprosy. N. Engl. J. Med..

[bb0325] Zhou H. (2011). Temporal expression of mutant LRRK2 in adult rats impairs dopamine reuptake. Int. J. Biol. Sci..

[bb0330] Zimprich A. (2004). Mutations in LRRK2 cause autosomal-dominant parkinsonism with pleomorphic pathology. Neuron.

