# Supplementary material for: Genetic and pharmacological evidence that G2019S LRRK2 confers a hyperkinetic phenotype, resistant to motor decline associated with aging
Source: Neurobiol Dis. 2014 Nov;71:62–73. doi: 10.1016/j.nbd.2014.07.013 (PMC4194318; doi:10.1016/j.nbd.2014.07.013)
Supplement: Supplementary file 1 — Table S1. Motor performances of WT mice used in the study. Figure S1. Motor performances in aged-matched cohorts of G2019S KI and WT mice. Figure S2. H-1152 in 6-month-old mice. Figure S3. Rotarod performance in 12-month-old mice treated with H-1152. Figure S4. H-1152 in 15-month-old mice. Figure S5. H-1152 on pSer935 LRRK2 at 90 and 360 min after administration. Figure S6. Rotarod performance in 12-month-old mice treated with Nov-LRRK2-11. Figure S7. Blood and brain levels of Nov-LRRK2-11. Figure S8. pSer935 LRRK2 and LRRK2 protein levelsin striatum and cerebral cortex. [file mmc1.docx]

**Genetic and pharmacological evidence that G2019S LRRK2 improves motor performance and delays motor decline associated with aging**

^1^Francesco Longo, ^2^Isabella Russo, ^3^Derya R. Shimshek, ^2^Elisa Greggio, ^1^Michele Morari

*^1^Department of Medical Sciences, Section of Pharmacology, and National Institute of Neuroscience, University of Ferrara, via Fossato di Mortara 17-19, 44121 Ferrara, Italy.*

*^2^Department of Biology, University of Padova, Via Ugo Bassi 58/B, 35131 Padova, Italy. ^3^Department of Neuroscience, Novartis Institutes for BioMedical Research, Novartis Pharma AG, 4002 Basel, Switzerland.*

**Supplementary Material**

**Table S1**

**Figures S1-S8 with legends**

**Table S1**. A comparison between motor performances of wild-type mice used in the study, i.e. G2019S LRRK2 knock-in (KI) littermates (WT KI) and D1994S LRRK2 kinase-dead (KD) littermates (WT KD). Data are expressed as absolute values and are means ± SEM.

|  |  | **3** | **6** | **9** | **15** | **19** |
| --- | --- | --- | --- | --- | --- | --- |
|  |  |  |  |  |  |  |
| **WT KI** | Bar (sec) | 12.5 ± 2.9 | 17.4 ± 2.7 | 29.3 ± 3.2 | 34.2 ± 2.2 | 33.7 ± 2.3 |
|  | Drag (steps) | 17.7 ± 0.7 | 10.9 ± 0.7 | 10.5 ± 0.8 | 6.5 ± 1.1 | 3.4 ± 0.3 |
|  | Rotarod (sec) | 551.8 ± 49.5* | 646.0 ± 40.3* | 888.1 ± 37.7 | 691.9 ± 65.6 | 934.1 ± 40.2 |
|  | Distance (m) | nt | nt | nt | 64.6 ± 5.4 | nt |
|  | Immobility (sec) | nt | nt | nt | 656.3 ± 95.1 | nt |
|  |  |  |  |  |  |  |
| **WT KD** | Bar (sec) | 8.5 ± 1.0 | 16.9 ± 2.2 | 33.8 ± 3.4 | 34.3 ± 3.0 | nt |
|  | Drag (steps) | 16.2 ± 1.7 | 12.7 ± 0.4 | 8.5 ± 0.3 | 7.9 ± 0.6 |  |
|  | Rotarod (sec) | 752.3 ± 39.1 | 913.9 ± 47.4 | 1075.1 ± 90.1 | 824.4 ± 44.2 |  |
|  | Distance (m) | nt | nt | nt | 78.8 ± 7.9 | nt |
|  | Immobility (sec) | nt | nt | nt | 445.0 ± 77.8 | nt |
|  |  |  |  |  |  |  |

nt = not tested

*P<0.05, different from WT KD (Student t-test, two-tailed for unpaired data)

Figure S1

Figure S2

Figure S3

Figure S4


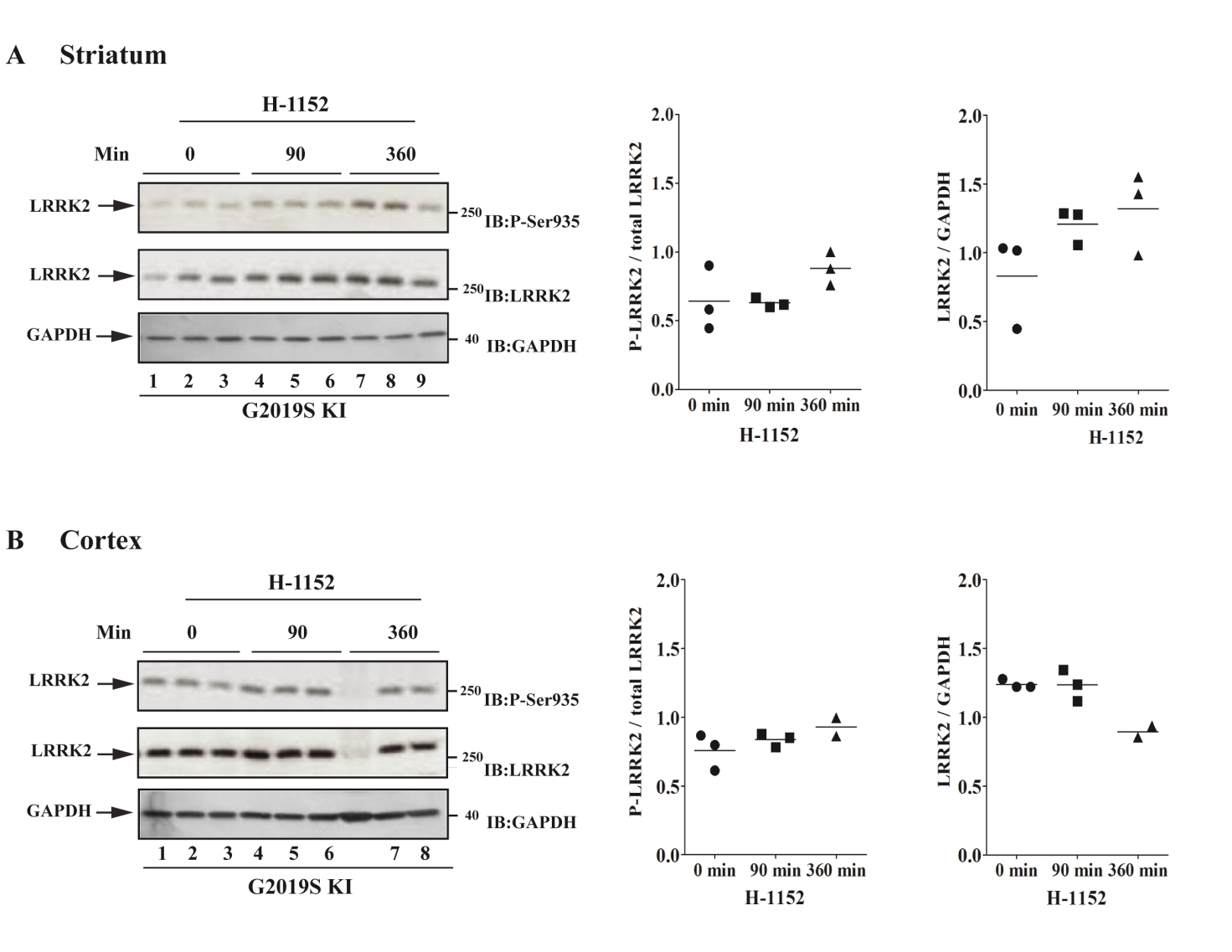


Figure S5

Figure S6





Figure S7





Figure S8

**Figure legends**

**Figure S1.** LRRK2 G2019S knock-in (G2019S KI) mice showed greater motor performances than wild-type littermates (WT). Motor performance was evaluated in age-matched cohorts of 3, 10, 14 and 18 month-old G2019S KI and WT mice, using the bar (A), drag (B), rotarod (C) and open field (D-E) tests. Motor activity was expressed as immobility time (sec; A, D), number of steps (B), time on rod (sec; C), and total distance travelled (m; E). Data are means ± SEM of 6-8 mice per group, and were analyzed using the Student t-test two-tailed for unpaired data.

°P<0.05,°°P<0.01 different from age-matched WT

**Figure S2**. The LRRK2 kinase inhibitor H-1152 reversed motor phenotype in G2019S knock-in (G2019S KI) mice being ineffective in wild-type littermates (WT). H-1152 was administered at 0.1 and 1 mg/kg (i.p.) in 6 month-old mice, and motor activity assessed using the bar (A), drag (B) and rotarod (C) tests, before (time 0; basal values) and 30 min after drug administration. Data are means ± SEM of 8 mice per group, and were analyzed using one-way RM ANOVA followed by the Newman-Keuls test for multiple comparisons.

**P<0.01 different from basal values

**Figure S3.** Time-course of the rotarod performance of 12 month-old G2019S knock-in (G2019S KI) mice and wild-type littermates (WT) treated with the LRRK2 kinase inhibitor H-1152. H-1152 was administered at 1 mg/Kg (i.p.), and motor activity assessed using the rotarod test, before (time 0; basal values) and 30, 90, 180, 360 min, and 24 hrs after drug administration. Data are means ± SEM of 6-8 mice per group, and were analysed using one-way ANOVA followed by the Newman-Keuls test for multiple comparisons.

*P<0.05 ,**P<0.01 different from basal values

**Figure S4**. Time-course of the motor effect of the LRRK2 kinase inhibitor H-1152 in 15 month-old G2019S knock-in (G2019S KI) and wild-type (WT) mice. H-1152 was administered at 1 mg/Kg (i.p.) and motor activity assessed using the bar (A), drag (B) and rotarod (C) test, before (time 0; basal values) and 30, 90, 180, 360 min, and 24 hrs after drug administration. Data are means ± SEM of 6 mice per group, and were analysed using one-way ANOVA followed by the Newman-Keuls test for multiple comparisons.

*P<0.05 ,**P<0.01 different from basal values

**Figure S5.** The LRRK2 kinase inhibitor H-1152 did not affect phosphorylation of LRRK2 at Ser935 *ex vivo* at 90 and 360 min after administration. Twelve month-old LRRK2 G2019S knock-in (G2019S KI) mice were treated with H-1152 (1 mg/Kg, i.p.), and sacrificed 90 or 360 min later. LRRK2 phosphorylation was measured *ex vivo* in the striatum (A) and cerebral cortex (B), before (time 0; T0), 90 min or 360 min after H-1152 administration. Results are the mean ± SEM of 3 mice per group, and were analysed using one-way ANOVA followed by the Newman Keuls test.

**Figure S6**. Time-course of the rotarod performance of 12 month-old G2019S knock-in (G2019S KI) mice and wild-type littermates (WT) treated with the LRRK2 kinase inhibitor Nov-LRRK2-11. Nov-LRRK2-11 was administered at 1 and 10 mg/kg (i.p.), and motor activity assessed using the rotarod test, before (time 0; basal values) and after (15, 75, 165, 360 min, 24 hrs) drug administration. Data are means ± SEM of 6-8 mice per group, and were analyzed using one-way RM ANOVA followed by the Newman-Keuls test for multiple comparisons.

*P<0.05 ,**P<0.01 different from basal values

**Figure S7**. Pharmacokinetic study showing blood and brain concentrations of Nov-LRRK2-11 after oral administration (by gavage) of the 3 mg/Kg dose. Each time-point represents the mean ± SEM of 3 mice.

**Figure S8**. Phosphorylation of LRRK2 at Ser935 and LRRK2 protein levels in the striatum (A) and cerebral cortex (B) of 12 month-old G2019S KI mice, D1994S KD mice, and respective WT littermates. Data are means ± SEM of 3 mice per genotype, and were analyzed using the Student t-test two-tailed for unpaired data.

*P<0.05 different from WT
